# Hippocampal abnormalities and sudden childhood death

**DOI:** 10.1007/s12024-016-9770-4

**Published:** 2016-03-30

**Authors:** Colin Smith

**Affiliations:** Department of Academic Neuropathology, University of Edinburgh, CCBS, Chancellor’s Building, Little France, Edinburgh, EH16 4SB UK

Sudden unexpected death in childhood (SUDC) is a devastating event for a family, and determining the cause of death can be a significant challenge for the pathologist. The actual incidence of both SUDC and sudden unexpected death in infancy (SUDI) is difficult to ascertain, particularly due to inconsistency with regard to definition and completion of death certification, and there are few reliable datasets [1]. Even the Centers for Disease Control and Prevention (CDC) data focuses predominantly on SUDI and sudden infant death syndrome (SIDS), with SUDC not being fully represented.

The San Diego SUDC research project was established approximately 15 years ago in an attempt to better understand the pathophysiological causes of SUDC. Hefti et al. [2] present data from this database, accrued 1999–2011. Of the referred 151 cases, 121 fulfilled the criteria of SUDC, as defined by the research project. When this group were compared to a group of explained childhood deaths, which included cardiac channelopathies, infections, and seizures, there was a significant overexpression of deaths during sleep periods and febrile convulsions within the SUDC group. Where appropriate histology was available almost 50 % of SUDC and unexplained cases showed hippocampal abnormalities. These hippocampal abnormalities are presented in greater detail in a subsequent paper [3], which in itself builds on an initial report by this group describing an entity labeled as hippocampal maldevelopment associated with sudden death (HMASD) [4]. The current paper presents data from a larger study population, and has suggested additional groups in addition to HMSAD, including SUDC with a febrile seizure phenotype (but no hippocampal maldevelopment) (SUDC-FS) and SUDC (without febrile seizure or hippocampal maldevelopment).

The high incidence of febrile seizures and death during sleeping periods has been reported by other groups [5], but to date the histopathological changes underlying HMSAD have only been documented by the Boston/San Diego group. In many cases the pathological changes may be very subtle (such as single ectopic granule cells) or subjective (hyperconvolution, focal lack of granule cells) and potentially could be missed by an inexperienced neuropathologist.

These publications are important additions to the literature of SUDC, and are an important epidemiological and pathological validation of the San Diego project. How should this now move forward? In my experience hippocampal abnormalities can be seen in SUDC cases. However, it is now for other centers to validate this data. In particular how reproducible are the changes associated with HMSAD, and are they seen with same incidence described in these papers? Due to the rarity of SUDC this may require a multi-center approach. My hope is that the current papers will define what is meant neuropathologically by HMSAD, and that neuropathologists with a significant pediatric workload will look for these changes and record them, however subtle. As important is the documentation of possible HMSAD neuropathology where there is a clear explanation for the cause of death—is HMSAD incidental rather than causal, or are some maldevelopmental changes more consistent with SUDC than others? When multi-center reproducible data exists, the basic biological research can begin to try and understand how these maldevelopmental changes can result in such devastating consequences.

Finally I must congratulate the vision of those who established the San Diego SUDC research project. It is only through such endeavors that our understanding of SUDC will advance.
